# Feasibility of Cerebellar Stimulation for the Treatment of Post-Stroke Dysphagia

**DOI:** 10.1007/s12311-025-01823-0

**Published:** 2025-03-26

**Authors:** Gwenllian Wilkinson, Ayodele Sasegbon, Craig J. Smith, Philip M. Bath, Shaheen Hamdy

**Affiliations:** 1 Stroke Trials Unit, Mental Health & Clinical Neuroscience, University of Nottingham, Nottingham, UK; 2 Nottingham University Hospitals NHS Trust, Stroke, Nottingham, UK; 3 Gastrointestinal (GI) Sciences, Division of Diabetes, Endocrinology and Gastroenterology, School of Medical Sciences, University of Manchester, Salford Royal Hospital (Part of the Manchester Academic Health Sciences Centre (MAHSC)), Salford, UK; 4 Manchester Centre for Clinical Neurosciences, Geoffrey Jefferson Brain Research Centre, Salford Royal Hospital, Northern Care Alliance NHS Trust, Salford, UK; 5 Division of Cardiovascular Sciences, University of Manchester, Manchester Academic Health Sciences Centre (MAHSC), Manchester, UK

**Keywords:** Cerebellum, Stroke, Neurostimulation, Oropharyngeal dysphagia, Transcranial magnetic stimulation, Repetitive

## Abstract

Post-stroke dysphagia (PSD) is common and associated with poor outcomes. We aimed to explore the feasibility, safety, and proof of concept of cerebellar rTMS in patients with sub-acute PSD. We intended to recruit 48 participants with PSD. Randomised to: (i) sham treatment twice-daily for five days, (ii) cerebellar rTMS daily for three days, and (iii) cerebellar rTMS twice-daily for five days (1:1:1). Participants were blinded to treatment group. Primary outcomes were feasibility, safety, and functional outcome intake scale (FOIS), dysphagia severity rating scale (DSRS), and feeding status scale (FSS) at two weeks. However, due to lower-than-expected enrolment, the active rTMS groups were combined. We recruited 14 participants in total, (mean 68 years, 57% female). Due to the time-limited funding period, recruitment was adversely affected by the COVID-19 pandemic. DSRS and FSS trended lower in the combined active rTMS groups at two weeks, i.e. less swallowing impairment. However, at death/discharge FOIS was higher/better (mean, (standard deviation)), 4.0 (2.1) vs. 1.8 (1.0) (*p* = 0.032) with active TMS, with trends to lower/better DSRS and FSS. There was no difference in the acceptability of treatment between groups. High-intensity (*n* = 5) vs. low-intensity (*n* = 5) cerebellar rTMS was associated with lower DSRS 3.0 (1.4) vs. 9.4 (2.7) and FSS 0.6 (0.5) vs. 1.6 (0.5) at 2 weeks, and DSRS 3.0 (1.4) vs. 9.0 (3.7) at hospital discharge or death. Cerebellar rTMS is a feasible ward-based treatment for reducing swallowing impairment. Although enrolment was lower than desired, there was evidence for proof of concept, particularly for high-intensity cerebellar rTMS. Larger studies are warranted.

## Introduction

Post-stroke dysphagia (PSD) is associated with poorer outcomes in relation to mortality, independence, and health economics [1]. Whilst there have been a number of guidelines in this area, there is no consensus as to which treatments are most likely to be effective [2]. Current treatment approaches rely on altering the consistency and textures of fluids or foods, swallowing rehabilitation guided by speech and language therapists, or the use of feeding tubes such as percutaneous gastrostomies and nasogastric tubes [3]. Repetitive transcranial magnetic resonance stimulation (rTMS) is a promising technique that utilises focused electromagnetic pulses to alter the activity of neurones within specific regions of the brain. Meta-analyses of rTMS applied over the areas of the primary motor cortices which correspond with the muscles used during the process of swallowing (tongue, pharynx, etc.) have shown that cortical rTMS improves swallowing function following hemispheric strokes [2, 4–8]. However, the cortical rTMS studies in these meta-analyses are heterogeneous, and, at present, there is no consensus as to optimal rTMS stimulation parameters. Most of the rTMS brain stimulation studies have applied stimulation to either undamaged or damaged sides of the brain unilaterally, bilaterally or more recently to the cerebellum [9]. However, there is no standardisation as to the number of sessions of rTMS in the post stroke period or the duration of followup [9]. Nonetheless, despite these drawbacks, the clinical potential of a mobile, non-invasive treatment for PSD is appealing to patients, researchers, and clinicians. More research is required to establish the degree and duration of the therapeutic effect of rTMS [10].

Volitional swallowing (saliva/dry and bolus swallows) activates a complex interplay of neurological areas with involvement from the primary motor cortex, somatosensory cortex (S1 and S2), supplementary motor area, premotor cortex, posterior parietal cortex, cingulate gyrus, inferior frontal gyrus, the cerebellum, the insular cortex, auditory cortex, corpus callosum, and the basal ganglia and thalamus [11]. The cerebellum modulates motor commands in response to changes in body position or applied load to muscles, which would include the oropharyngeal response to a presented bolus, via input from vestibular receptors and proprioceptors. This coordinating mechanism may explain why there is increased activity in the cerebellum during swallowing [12], and why rTMS applied to the cerebellum has been shown to activate motor responses in the pharynx [13, 14]. Drawing on the neurophysiological foundation provided by cortical rTMS, cerebellar rTMS was developed as a treatment for neurogenic dysphagia [13–17]. It has also been demonstrated that volitional swallowing involves cerebellar activation [15] and stimulation of the cerebellum excites the cortico-bulbar pathways to the pharynx [13]. In healthy participant neurophysiological studies, it has been found that cerebellar rTMS delivered at a frequency of 10 Hz resulted in prolonged cortico-pharyngeal excitability (measured by motor evoked potential [MEP]), which lasted at least 30 min post intervention [14]. This dose regimen was found to reverse the effects of a time limited cortical hemispheric lesion, ‘virtual-lesion’, in healthy participants [17]. Utilising cerebellar rTMS protocols developed in healthy participants [14, 18], unihemispheric and bihemispheric cerebellar rTMS has been shown to improve PSD in a small number of studies following brainstem [19] and hemispheric strokes [20, 21]. However, gaps in our understanding of the effects of cerebellar rTMS on PSD remain including the number of rTMS pulses needed to optimally stimulate the cerebellum and whether there is a relationship between stimulation intensity and neuronal excitability [22–24].

Here, we report a phase II dose comparison trial of cerebellar rTMS for the treatment of PSD exploring feasibility, safety, and proof of concept.

### Patients and Methods

The published protocol for the trial (see study 2^25^) includes the full inclusion and exclusion criteria, interventions, outcomes, and sample size discussion. In brief, adults with PSD within 12 weeks of stroke and a dysphagia severity rating scale (DSRS [25]) score ≥ 7 were recruited from the acute stroke unit at Nottingham University Hospitals NHS Trust (Queens Medical Centre campus) from September 2021 to June 2022. Patients were identified for screening if they were referred to the stroke speech and language therapy (SLT) team for swallowing management. Screening was based on clinical evaluation by the SLT team who use the DSRS as a clinical outcome measure. Patients able to provide informed consent and those unable to do so were eligible for inclusion in the study. In cases where patients were unable to provide consent, consent was obtained via consultee declaration after meeting with the patient’s next of kin and clinical team. While the preference was to obtain informed consent from all study participants, ethical approval was obtained for consultee assent, due to the fact that in the post stroke period, patients may be too unwell to give full informed consent. Potential issues in the immediate post stroke period include the inability to retain information, difficulty weighing complex decisions, or problems fulsomely conversing/communicating with the research team. Due to the relatively low risk of cerebellar rTMS, a technique which has not had any reported serious adverse effects in the literature, the known deleterious effects of post stroke dysphagia, and the potential swallowing benefits of study enrolment, ethical approval was given to discuss the study with both the patient’s clinical team and next of kin. Only if both parties felt it was in the patient’s best interest would the study then proceed, a process utilised in other acutely unwell patient studies [26].

Ethical Approval was granted by Greater Manchester South NHS research ethics committee (18/NM/0232, 03 Aug 2018). The study was registered on clinicaltrials.gov (NCT03274947) and funded by the Medical Research Council (grant no. MR/P006183/1, start and end dates 07/07/2017-31/08/2022).

### Treatment

Participants were randomised 1:1:1 to one of three groups via an encrypted digital randomisation web-based algorithm. Participants were blinded to group allocation. Furthermore, group allocation was concealed from researchers to prevent researchers from knowing ahead of time which participant was allocated to which group. This was done by the web-based algorithm generating the allocated treatment group immediately prior to each patient’s therapeutic session. The three treatment groups comprised: (i) Low-intensity rTMS applied once daily for 3 days; (ii) High-intensity rTMS group applied twice daily for 5 consecutive days; and (iii) Sham group twice daily for 5 consecutive days with the coil held at a 90-degree angle from the skull so that the pulses could be heard but stimulation was not applied. RTMS was applied as per the currently established optimal frequency of 10 Hz for 250 pulses, as per previous cortical rTMS optimisation studies, (Super-rapid, Magstim Co., Whitland, UK) at an intensity of 90% of the thenar resting motor threshold applied via a figure of eight magnetic coil with an outer diameter of 70 mm to the contralesional cerebellar hemisphere. The location of the coil was based on measurements to the left or right of the inion of the skull, as previously described [13, 27]. Treatment was applied at the bedside, on each participant’s ward.

### Outcomes

The original primary clinical outcome was the penetration aspiration scale (PAS) score derived from videofluoroscopic (VFS) examination [28]. However, patient recruitment commenced in 2019 shortly before the COVID-19 pandemic stopped all non-COVID related clinical research activity. Even when some research was allowed to recommence severe limitations were put in place due to concerns about aerosol generation and moving patients around hospitals. Trial funding then ended in 2022. Hence, the primary clinical outcome was changed following a protocol amendment to the previous secondary outcomes of swallowing impairment, measured using three scales. The functional oral intake scale (FOIS [29]) is a validated 7-point scale assessing nutritional intake including enteral feeding, regular and modified oral intake; the scale ranges from 1 = nothing by mouth to 7 = total oral diet with no restrictions [29]. The DSRS is a validated 12-point scale calculated from the sum of three subscales covering diet, fluids, and supervision [24, 25]. The scale captures enteral feeding and/or oral intake of “regular” and modified food/drink and has been adapted to use the international dysphagia diet standardisation initiative (IDDSI) scale [30]. The feeding status scale (FSS [31]) assesses how patients are taking in fluids/food in the presence of dysphagia and ranges from 0 = normal to 6 = death. Outcome measures were assessed at baseline, two weeks post intervention, and at discharge/death (or 30 days post intervention for ongoing inpatients). Outcomes were taken from clinical records based on routine clinical evaluation by SLTs who were not aware of group allocation.

### Sample Size

The original sample size of 48 was estimated on the basis of anticipated PAS scores across the three groups [28]. However, the FOIS, DSRS, and FSS were uprated from secondary to primary outcomes due to unavoidable COVID-19 related research restrictions. This approach has been used in previous neuromodulation studies [32]. However, it was recognised that while clinical swallowing assessment tools are useful and given valuable information, they are less objective and sensitive than instrumental examinations. These restrictions also meant fewer participants were recruited than anticipated potentially limiting the generalisability of the study findings.

### Data Analysis

Data are number (%) or mean (standard deviation). Originally, we planned to compare the three groups separately but the limited number of participants due to COVID-19 led to us combining the low- and high-intensity groups (groups A and B in the protocol paper [28]) in most analyses. Comparison of treatment with rTMS (combined low- and high-intensity groups) vs. sham rTMS was performed using multiple linear regression with adjustment for age and baseline stroke impairment using the National Institutes of Health stroke scale (NIHSS); comparisons were performed via unpooled t-test at two time points: (i) two weeks post randomisation, (ii) death or discharge. Low- and high- intensity groups were compared at the same time points. Statistical significance was set at *p* < 0.05; 95% confidence intervals are shown. Statistical analysis was performed using SPSS version 26 (IBM SPSS Statistics for Windows, IBM Corp., Armonk, NY, USA).

## Results

**Fig. 1:**
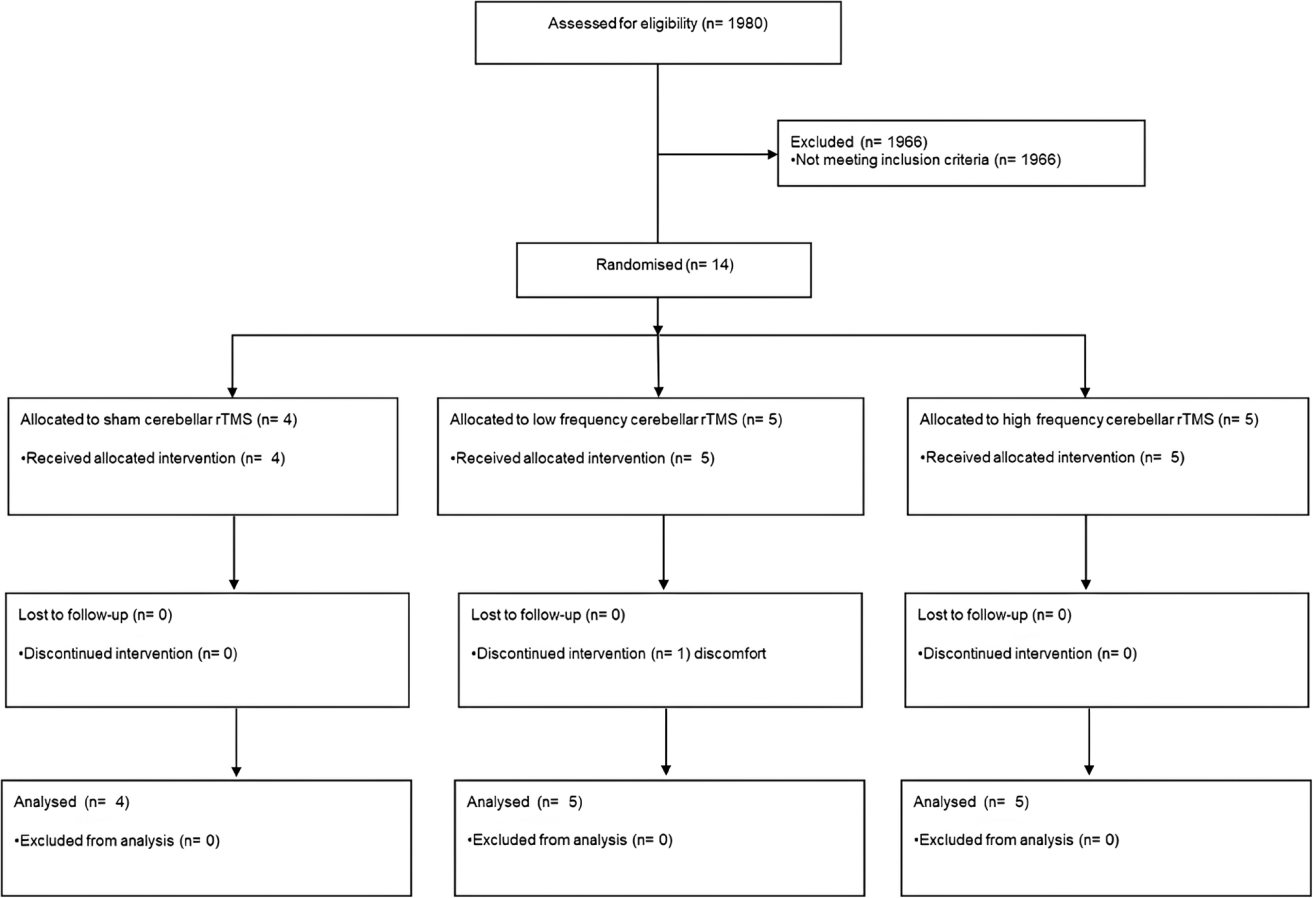
CONSORT flow diagram of recruitment

All admissions to the stroke unit at Nottingham University Trust hospitals between August and October 2022 were screened for eligibility (Fig. 1). Of 1980 screened for eligibility, 1966 were excluded. Fourteen participants were recruited in total (sham 4; low-intensity 5; high-intensity 5) recruitment ended at the end of the funded trial period; one participant in the low-intensity group withdrew from treatment before completion but agreed to ongoing data collection. The mean age was 68 (19) with 8 (57%) women, baseline NIHSS 9.9 (6.5), and 8 (57%) ischaemic stroke (Table 1). Baseline swallowing impairment was FOIS 1.6 (0.63), DSRS 10 (1.9), and FSS 3.1 (0.5); in *post hoc* analyses, significant differences were present for FOIS to be higher (ANOVA F = 8.84, *p* < 0.01) and DSRS lower (ANOVA F = 4.09, *p* = 0.047) with rTMS, highest in the high-intensity group and lowest in the sham group (Table 1). However, there were no significant differences at baseline for FOIS, DSRS, and FSS scores between the high and low intensity rTMS groups (T = 1.888, 1.373, 0.994, *p* = 0.095, 0.207, and 0.349).

**Table 1:** Baseline characteristics. Data are number (%) or mean (standard deviation)

	All (*N*)	High intensity group	Low intensity group	Sham group
Participants	14	5	5	4
Age (years)	68.0 (19.2)	65.4 (19.0)	71.6 (23.4)	66.8 (18.9)
Sex, female (%)	8 (57)	3 (60)	3 (60)	2 (50)
NIHSS (/42)	9.9 (6.5)	9.4 (6.9)	9.4 (6.3)	11.3 (7.8)
Stroke type, ischaemic (%)	8 (57.1)	3 (60)	3 (60)	2 (50)
Anterior circulation (%)	11 (78.6)	4 (80)	4 (80)	3 (75)
OTR (days)	15.0 (10.0)	13.0 (8.0)	12.0 (9.0)	22.0 (11.0)
FOIS	1.64 (0.63)	2.20 (0.45)	1.60 (0.55)	1.00 (0.00)
DSRS	10.6 (1.9)	9.00 (1.58)	10.6 (2.07)	12.0 (0.0)
FSS	3.1 (0.5)	3.0 (0.0)	3.4 (0.9)	3.0 (0.0)

DSRS: dysphagia severity rating scale; FOIS: functional oral intake scale; FSS: feeding status scale; OTR: onset to randomisation

At two-weeks post intervention, the combined rTMS groups had adjusted non-significant tendencies to reduced swallowing impairment, i.e. higher FOIS/lower DSRS, and FSS, in comparison with the sham group (Table 2). Although the same pattern was seen at discharge (at a median of 39.0 [IQR 22.5–48.0] days) or hospital death, only the FOIS scores in the combined rTMS group were significantly higher/better than sham, 4.00 (2.11) vs. 1.75 (0.96), difference 2.53 (95% CI 0.28, 4.78) (2p = 0.032) (Table 2). Comparison between the two active groups indicated a benefit of high- over low-intensity cerebellar rTMS at two weeks for DSRS: 3.0 (1.4) vs. 9.4 (2.7), difference in mean − 8.0 (-14.0, -2.0); and FSS (0.6 (0.5) vs. 1.6 (0.5), difference in mean − 1.7 (-3.1, -0.3 (Table 3). This was also seen in DSRS scores at hospital discharge or death: 3.0 (1.4) vs. 9.0 (3.7), difference in mean − 7.5 (-14.9, -0.1).

**Table 2:** Comparison of swallowing scales between active and Sham groups at 2 weeks and discharge or death. Data are number (%) or adjusted mean (standard deviation). Comparison by multiple linear regression adjusted for age and baseline NIHSS showing point estimate and 95% confidence intervals

	*N*	All	*N*	Combined active groups	*N*	Sham	Difference in means (95% CI)	2p
*2 weeks*								
FOIS	14	3.00 (1.62)	10	3.40 (1.71)	4	2.00 (0.82)	1.6 (-0.5, 3.6)	0.12
DSRS	14	7.29 (3.87)	10	6.2 (3.9)	4	10 (2.2)	-4.2 (-8.7, 0.3)	0.065
FSS	14	2.36 (0.84)	10	2.1 (0.74)	4	3.0 (0.82)	-0.9 (-2.0, 0.2)	0.083
*Discharge or death*								
FOIS	14	3.36 (2.10)	10	4.00 (2.11)	4	1.75 (0.96)	2.5 (0.3, 4.8)	0.032
DSRS	14	7.07 (4.27)	10	6.00 (4.11)	4	9.75 (3.86)	-4.3 (-9.3, 0.7)	0.086
FSS	14	2.50 (1.02)	10	2.2 (0.92)	4	3.3 (0.96)	-1.1 (-2.4, 0.1)	0.073

DSRS: dysphagia severity rating scale; FOIS: functional oral intake scale; FSS: feeding status scale

**Table 3:** Comparison of swallowing scales between high intensity active group and low intensity active group at 2 weeks and discharge or death. Data are number (%) or adjusted mean (standard deviation). Comparison by multiple linear regression adjusted for age and baseline NIHSS showing point estimate and 95% confidence intervals

	*N*	All	*N*	High intensity	*N*	Low intensity	Difference in means (95% CI)	*p* value
*2 weeks*								
FOIS	10	3.4 (1.7)	5	4.4 (1.3)	5	2.4 (1.5)	2.8 (-1.3, 6.8)	0.15
DSRS	10	6.2 (3.9)	5	3.0 (1.4)	5	9.4 (2.7)	-8.0 (-14.0, -2.0)	0.02
FSS	10	2.1 (0.7)	5	0.6 (0.5)	5	1.6 (0.5)	-1.7 (-3.1, -0.3)	0.02
*Discharge or death*								
FOIS	10	3.4 (2.1)	5	5.2 (1.5)	5	2.8 (2.0)	2.8 (-2.0, 7.5)	0.20
DSRS	10	7.1 (4.3)	5	3.0 (1.4)	5	9.0 (3.7)	-7.5 (-14.9, -0.1)	0.05
FSS	10	2.2 (0.9)	5	0.6 (0.5)	5	1.8 (0.8)	-1.4 (-3.5, 0.6)	0.14

DSRS: dysphagia severity rating scale; FOIS: functional oral intake scale; FSS: feeding status scale

No serious adverse device effects occurred. Two serious adverse events (SAE) occurred by discharge or day 30, one case each of fatal myocardial infarction and non-fatal stroke recurrence. Neither SAE occurred during the treatment window nor were thought to be related to treatment (Table 4). One adverse device effect (ADE) occurred during the treatment window: a participant aged 32 who was post-hemicraniectomy withdrew from treatment due to discomfort during stimulation. The SAEs and ADE occurred in the low-intensity group. The low event rates precluded statistical comparisons.

**Table 4:** Serious adverse events (SAE) at day 30/discharge across high intensity, low intensity and Sham groups. Data are number (%)

	All	High-intensity	Low-intensity	Sham
Chest infection/pneumonia	0 (0)	0 (0)	0 (0)	0 (0)
In hospital mortality	1 (7.1)	0 (0)	1 (20.0)	0 (0)
Death by end of the trial	0 (0)	0 (0)	0 (0)	0 (0)
Serious adverse event	2 (14.3)	0 (0)	2 (40.0)	0 (0)
Serious adverse device event	0 (0)	0 (0)	0 (0)	0 (0)
Adverse device effect	1 (7.1)	0 (0)	1 (20.0)	0 (0)
Withdrew from treatment	1 (7.1)	0 (0)	1 (20.0)	0 (0)
Withdrew from follow-up	0 (0)	0 (0)	0 (0)	0 (0)

## Discussion

We found that contralesional cerebellar rTMS was feasible to apply, safe, and showed the potential for improving swallowing ability in patients with acute PSD. Conducting cerebellar rTMS on a stroke unit was an unobtrusive and simple treatment for patients with PSD. Furthermore, this is the first cerebellar rTMS study which has used functional clinical swallowing scales to assess post-treatment swallowing improvement compared to PAS scores. This approach is less arduous than VFS PAS based approaches and more in keeping with routine clinical practice by speech and language therapists. The differences in means between rTMS and sham for FOIS, DSRS, and FSS were consistently in a direction suggesting potential benefit, and significantly so for FOIS at discharge/death. Contextualising our study results with the existing literature, cerebellar rTMS can be said to result in both measurable improvements in PAS scores [21, 33], but also observable clinical improvements in swallowing ability. Although the sham protocol of angling the rTMS coil away from the scalp has been used extensively in rTMS research for PSD and other conditions [34], analysis of potential placebo effects arising from the auditory and tactile sensations of rTMS has not been conducted and should be considered. Going forward, significant effort must be made to standardise cerebellar treatment protocols through a process of neurophysiological studies in healthy participants and subsequent head to head therapeutic comparisons in patients with PSD.

Multiple studies of cortical rTMS have been reported after stroke and meta-analyses of those done as a potential treatment for PSD have suggested a beneficial effect [2, 4–8]. RTMS applied to the cerebellum has been shown to activate motor responses in the pharynx [13, 14], and its intensity and method of administration have been adjusted to further optimise its ability to enhance brain excitability and improve swallowing function in the intact human brain [13–17, 28]. Other studies have demonstrated its effectiveness at improving PSD caused by hemispheric or brain stem strokes utilising unilateral and/or bilateral stimulation [19, 21, 35, 36]. Cerebellar rTMS may also have advantages compared to cortical rTMS as it does not need accurate targeting of the cerebellum and can be applied using anatomical landmarks [18, 37]. Furthermore, cerebellar rTMS does not have any recognised propensity to induce kindling or seizure activity in patients with neurological conditions [38]. Furthermore, the regimen applied is shorter and less time consuming than standard 5 Hz cortical rTMS. As such, cerebellar rTMS has the potential to be a feasible alternative to other forms of non-invasive brain stimulation.

Unfortunately, the trial had to adapt to two significant challenges. First, the Research Ethics Committee initially would not allow consultee assent on the grounds that participants may not obtain benefit from phase-2 feasibility trials. Although a common perspective, we considered this overly cautious in a population of patients with a severe condition such as post-stroke dysphagia. Our participants had a baseline NIHSS score of 9.9 at a mean of 15 days from stroke onset; this score is considered moderate-to-severe in the first few hours after stroke and so signifies very severe neurological impairment at 15 days. Unsurprisingly, few patients had the capacity to give their consent, and so most potential patients were screened out. Hence, we had to reapply for ethics committee approval to allow consultee assent; although this was given, it delayed then limited recruitment within our funding window. Assent was well received by the participants’ next of kin and there were no subsequent issues arising from the participants. Second, due to the limited funding period, the trial was severely affected and consequently restricted by COVID-19. Our hospital appropriately limited patient movements and restricted aerosol-generating procedures. We had originally planned to have a primary outcome based on VFS assessment of the penetration aspiration scale and the sample size was based on this. As a result, we had to limit our outcomes to clinical swallowing assessments. It is arguable that clearly observable clinical improvements in swallowing function are critical to improving dysphagia therapies, meaning our focus on clinical swallowing measures ensured the clinical relevance of the study. However, removing the objective outcomes of motor evoked potential amplitudes (MEPs) and VFS PAS assessments will have reduced the precision of our study findings and introduced a potential source of bias as clinical swallowing measures are more subjective. Furthermore, the sample size calculation was performed on previous instrumental outcomes rather than clinical ones. However, despite all these limitations, changing the study outcome measures allowed the study to continue despite COVID-19 restrictions and allowed the collection of more “real world” data in a way that has not been attempted before.

The low patient numbers and the necessity to combine both active treatment groups reduced the power of the experiment and will have had the effect of increasing the type II error rate of any statistical analysis. Therefore, true differences between groups have the potential to be lost. This fact must be borne in mind when cautiously interpreting the study data. Another limitation is the fact that patients were only followed up for a short period of time post cerebellar rTMS stimulation (weeks). Although this is often the case in studies of rTMS [9], often due to the desire to simplify the overall study design, PSD is potentially a long lasting condition meaning it is important to establish the durability of rTMS induced swallowing improvements. This is a weakness affecting the majority of current rTMS studies necessitating the need for longer follow-up in future studies. Lastly, it should be borne in mind that there were significant differences in the baseline measurements between the combined active and sham treatment groups. Despite robust attempts at randomisation, the patients in the combined active group were slightly less dysphagic than those in the sham group. Considering all these factors, the slightly reduced dysphagia severity in the combined active group may have made it more difficult to detect significant changes in that group. The fact that significant changes were noted despite these potential effects is therefore noteworthy.

In terms of adverse events, only one participant receiving active rTMS withdrew from treatment (but not follow-up) due to discomfort. However, in that specific case, it is possible that the partial lack of skull following their hemicraniectomy might have led to hypersensitivity and so discomfort.

## Conclusion

In summary, cerebellar rTMS was feasible to administer, appeared safe and proof of concept was supported. Despite the relatively small number of participants, which must be borne in mind during any interpretation of the data, our findings point towards cerebellar rTMS improving PSD with high intensity cerebellar rTMS having a greater positive effect than low intensity cerebellar rTMS. Further larger trials are now required to evaluate clinical effectiveness. These should be based on clinical rather than radiological or endoscopic assessments to reflect real-world practice. The effect of combining cerebellar rTMS with motor training could also be considered as part of gaining a better understanding of rehabilitation approaches to PSD.

## Data Availability

All data supporting the findings of this study are available within the paper.
